# 

**Published:** 1902-07

**Authors:** 


### BOOKS RECEIVED.

American Edition of Nothnagel's Encyclopedia, Vol. III. Diphtheria. By Wm. P. Northrup, M. D., of New York. Measles, Scarlet Fever and German Measles. By Professor Dr. Th. von Jurgensen, Professor of Medicine in the University of Tubingen. Edited, with additions. by William P. Northrup, M. D., Professor of Pediatrics in the University and Bellevue Medical College, New York. Handsome octavo, 672 pages, illustrated, including 24 full-page plates, three of them in colors. Philadelphia and London : W. B. Saunders & Co. 1902. [Cloth, $5.00 net ; Half-Morocco, $6.00 net.]

Diseases of the Nose, Pharynx and Ear. Bv Henry Gradle, M. D., Professor of Ophthalmology and Otology, Northwestern University Medical School, Chicago. Octavo of 547 pages, profusely illustrated, including two full-page plates in colors. Philadelphia and London : W. B. Saunders & Co. 1902. [Cloth, $3.50 net.]

The Practical Medicine Series of Year Books. Ten volumes. Issued monthly under the general editorial charge of Gustavus P. Head, M. D., Professor of Laryngology and Rhinology in the Chicago Post-Graduate Medical School. Volume VI. General Medicine. Edited by Frank Billings, M. S., M. D. Head of Medical Department and Dean of the Faculty of Rush Medical College, Chicago. With the Collaboration of S. C. Stanton, M. D. May, 1902 12mo, pp. 271. Chicago : The Year Book Publishers. [Price, $1.50. Entire series, $7.50.]

A Practical Treatise on Smallpox. Illustrated by colored photographs from life. By George Henry Fox, M. D., Consulting Dermatologist to the Health Department of New York City. With the Collaboration of S. D. Hubbard, M. D., S. Pollitzer, M. D. and J. H. Huddleston, M. D. In two parts, atlas form. Philadelphia and London : J. B. Lippincott Co. 1902. [Price, $3.00.]

The Neuroses of the Genitourinary System in the Male, with Sterility and Impotence. By Dr. R. Ultzmann, Professor of Genitourinary Diseases in the University of Vienna. Second edition. Revised, with notes and a supplementary article on Nervous Impotence, by the translator, Gardner W. Allen, M. D., Surgeon in the Genitourinary Department of the Boston Dispensary ; Instructor in Genitourinary Surgery in Tuft's Medical College Illustrated. 12mo, 198 pages. Philadelphia : F. B. Davis Company. 1902. [Price, $1.00 net, delivered.]

Practical Dietetics. With Special Reference to Diet in Disease By W. Gilman Thompson, M. D., Professor of Medicine in the Cornell University Medical College, New York City ; Visiting Physician to the Presbyterian and Bellevue Hospitals. 8vo, pp. 851. Second edition, enlarged and thoroughly revised. New York : D. Appleton & Company. 1902. [Price, $5 00.]

Minor Surgery and Bandaging. By Henry R. Wharton, M. D., Professor of Clinical Surgery in the Woman's Medical College, Surgeon to the Presbyterian Hospital, Philadelphia, etc. In one 12mo volume of 612 pages, with 509 illustrations, many of which are photographic. Fifth edition enlarged and thoroughly revised. Philadelphia and New York : Lea Brothers & Co. 1902. [Price, $3.00 net.]

The Artificial Feeding of Infants. By Charles F. Judson, M. D., Physician to the Medical Dispensary of the Children's Hospital, and J Claxton Gittings, M. D., Assistant Physician to the Medical Dispensary of the Children's Hospital. 12mo, pp. 368. Philadelphia: J. B. Lippincott Company. 1902. [Price, $2.00.]

Progressive Medicine, Vol. II,, June, 1902. A Quarterly Digest of Advances, Discoveries and Improvements in the Medical and Surgical Sciences. Edited by Hobart Amory Hare, M. D, Professor of Therapeutics and Materia Medica in the Jefferson Medical College of Philadelphia. Octavo, 440 pages, 28 illustrations. Philadelphia and New York: Lea Brothers & Co. [Per annum, in four cloth-bound volumes, $10.00. Per volume, $2.50, by express prepaid to any address.]

Transactions of the Southern Surgical and Gynecological Association. Fourteenth Annual Meeting held at Richmond, Va., November 12, 13 and 14, 1901. W. D. Haggard, M. D., Secretary. Published by the Association. 1902.

The Practitioner's Manual. A Condensed System of General Medical Diagnosis and Treatment. By Charles Warrenne Allen, M. D., Consulting Genitourinary Surgeon to the City (Charity) Hospital, Consulting Dermatologist to the Randall's Island Hospital, to the Hackensack Hospital, etc. Octavo, pp. 893. Second edition, revised and enlarged New York: William Wood & Company. 1902. [Price, muslin, $6.00; half-morocco, $7 00 net.]

A System of Physiological Therapeutics. A Practical Exposition of the methods, other than Drug-giving, useful in the prevention of Disease and in the Treatment of the Sick. Edited by Solomon Solis Cohen, A. M, M D., Professor of Medicine and Therapeutics in the Philadelphia Polyclinic; Lecturer on Clinical Medicine at Jefferson Medical College; Physician to the Philadelphia Hospital and to the Rush Hospital for Consumption, etc In eleven 8vo volumes. American, English, German and French authors. Volume IX. Hydrotherapy, Thermotherapy, Heliotherapy and Phototherapy. By Dr. Wilhelm Winternitz, Professor of Clinical Medicine in the University of Vienna; Director of the General Polyclinic in Vienna; Assisted by Dr. Alois Strasser, Instructor in Clinical Medicine at the University of Vienna, and Dr. B. Buxbaum, Chief Physician of the Hydrotherapeutic Institute in Vienna. And Balneology and Crounotherapy by Dr. E. Heinrich Kisch, Professor in the University of Prague; Physician at Marienbad, Spa. Translated by Augustus A. Eshner, M. D., Professor of Clinical Medicine in the Philadelphia Polyclinic. Illustrated. Philadelphia: P. Blakiston's Son & Co. 1902. [Price for the set complete, $22.00 net.]

A Manual of Otology. By Gorham Bacon, A. M., M. D., Professor of Otology in Cornell University Medical College, New York. With an introductory Chapter by Clarence J. Blake, M. D., Professor of Otology in Harvard Medical School, Boston. New (3d) edition. In one 12mo volume of 437 pages, with 120 engravings and 7 plates in colors and monochrome. Philadelphia and New York: Lea Brothers & Co, Publishers. [Price, cloth, $2.25 net.]

---

### LITERARY NOTES.

### Index to New Series Volume XLI.

### (Whole Number Volume LVII.)

### August, 1901–July, 1902

PAGE.

ABDOMINAL OPERATIONS, Rectal Feeding after . . . 437
Adenoids in Relation to Structural changes . . . . . . 732
Alcoholism, Insomnia of . . . . . 17
its Control and Cure in Sanatoriums . . . 880
Amblyopia, After Use of Jamaica Ginger . . . . . . . . . . 37
Anesthesia, Local . . . . . . 105
Anesthetic, Choice of for Children 173
Aneurism, Notes on Case of Abdominal . . . 121
Angina, Ulcero-membranous . 674
Antitoxin, Commercialism and Responsibility in Connection with . . . . 434
Laboratory, State . . 62
Apoplexy Following Trauma. 372
Appendicitis and the Vermiform Appendix . 257
Complicating Pregnancy, etc . . . 406
Asthenopia, Its Frequency in America. . . 117
Asthma, Treatment of . . . . . . 126
Ataxia, Locomotor. . . . . 128
Iodides in . . 125

BEAHAN, A. L., Hospitals in Smaller Towns . . . . 184
Blackham, George E., Ethics of Nursing . . . . . 251
Books Received; 80, 159, 239, 317, 392, 469, 546, 626, 706, 783, 857, 936
Breath, Fetor of . . . . . . . . 524
Brothers, Samuel F., Appendicitis Complicating Pregnancy, Labor and the Puerperium . . . . . 406
Brown, George L., Pruritus Ani . . 819
Buffalo Fresh Air Mission . . . . . 919
Society for the Prevention of Tuberculosis . . . . . . 918

CANCER LABORATORY Report of Buffalo State . . 569
Carbolic Acid, Its Use and Abuse . . . . . . 337
Cardiac Diseases, Anesthetics in. . . 127
Catarrh, Treatment of Nasal . . . . 440

PAGE.

Cecum, Removal of for Carcinoma. . 168
Chancre, The Unrecognised. . . . . 758
Charity, by Proxy . . . . . . 22
Chest, Penetrating Wounds of . . 270
Chorea, Etiology and Treatment of 510
Christian Science and the Coroner . 769
Circumcision, Infant . . . . . . . 891
Cities, A Tale of Two . . . . . . 128
Cleft Palate . . . . . . . . 909
Clinton, Marshall, Notes on Case of Abdominal Aneurism . . 121
Prostatic Hypertrophy . . 12
Coach Service, An Invalid . . . . . 138
Colitis in Children . . . . . . . . 38

**College and Hospital Notes:**

Albany Hospital. . . . . . . . 72
Alpha Omega Delta Fraternity, U. of B. . . . . . . 687
Brooks Memorial Hospital. . . . 686
Buffalo Consumption Hospital 773
Buffalo Hospital Sisters of Charity. . . . 453, 536, 773
College of Physicians and Surgeons of Baltimore . 686
Emergency Hospital Genito-urinary Dispensary . . . . 926
Fresh Air Mission Hospital. . . 71
German Hospital. . . . 536, 773
Marine Hospital for Buffalo, 454, 686
McGill University . . . 453
Medical Department, University of Buffalo . . . . 71, 309, 773
New Emergency Hospital . 536, 686
New York Orthopedic Dispensary and Hospital . . . . . 385
Riverside Hospital Dispensary . 926
State Hospitals, Inspection of by Governor Odell. . . . . . 71
State Hospital for Consumptives . . . 454, 686
St. Mary's Infant Asylum and Maternity Hospital . . . . . 687
Syracuse College of Medicine . 687
Syracuse University Summer School . . . . . . . 774
University of Buffalo Faculty Prizes . . . . . . . . . 774
University State of New York . 453

PAGE.
Comfort Stations . . . . . . . . . 61
Conklin, William L., Clinical vs. Bacteriological Diagnosis and Quarantine of Diphtheria . . . . . . . . 660
Removal of the Cecum for Carcinoma. . . . . 168
Connecticut Law and Narcotic and Alcoholic Patients . . . . . . . 614
Cornea, Pathology of Serpent Ulcer of . . . . . . . . . . . . . . 36

**Correspondence:**

Aloes and Hemorrhoids—John H. Grant . . . . . . . . 293
Collective Investigation Relating to Silver Nitrate Injections in Phthisis—Thomas J. Mays . . . . . . . . . 129
Examinations for U. S. Marine Hospital Service—Walter Wyman. . . . . . . . 837
Fire at the College of Physicians and Surgeons, Chicago—Frank B. Earle. . . . . 129
Knell of Sectarianism in Medicine—DeLancey Rochester. . 836
Maltine Company's Prizes—Charles C. Heuman. . . . 524
Medical Society, State of New York, Back Volumes of Transactions—F. C. Curtis. . . . . 294
Opening for Internes in State Hospitals — Frederick Peterson . . . . . . . . . . . 761
Pathologic Exhibit of American Medical Association — F. M. Jeffries . . . . . . . . . . . 760
Removal — Success and Gratitude—Martin H. Smith Co . . 835
Thirteenth International Medical Congress Transactions — A. Chanthard . . . . . 294
Vaccine Virus—Parke, Davis & Co. . . . . . . . . . . . . 441

Cott, George F., Non-surgical Treatment of Commonest Form of Deafness . . . . . . . . . . . . . 349
Cuba, Changes Wrought in . . . 848
Cystitis, Treatment of in Women. . 363
Obstinate . . . . . . . . . 587

DAGGETT, Byron H., Anatomy of the Kidneys . . . . . . 24
Davis, N. S., Banquet to. . . 299
Deafness, Non-surgical Treatment of Commonest Form of. . . . 349
Death, Rare Causes of Sudden in Children. . . . . . . . . . 37

PAGE.
Dentist and Surgeon, Relation Between . . 826
Diarrhea in Infants, Treatment of . 40
Diehl, Alfred E., A Case of Mycosis Fungoides . . . . . . . . . 658
Diphtheria, Clinical vs. Bacteriological Diagnosis of . 660
Complicating Measles. 675
Extraordinary Results in Treatment of . . . . 358
Diuretic, An Agreeable. . . . . 124
Dooley, Mr., on the Practice of Medicine . . . . . . . . . . 50
Dowd, J. Henry, Renal and Bladder Tuberculosis . . . . 482
The Two Glass Test 181
Treatment of Prostatic Disease by Hot Solution 884
Durrent, J. A., Successful Treatment of Obstinate Hiccough . . . . . 194

EPILEPSY, Operations for Traumatic . . . . . . . . . . . 818
Plea for Broader Treatment of. . . 161
and Infectious Diseases . 371
Salt Starvation in the Bromide Treatment of. . . . . 436
Expectoration in Street Cars. . . . 680
Explanation. . . . . . . . . . . . 240

FLOECKINGER, F. C., Local Anesthesia . . . 105
Forsyth, Edgar A., Deviation of the Nasal Septum 655
Fowler, George Ryerson, Suits for Surgical Service . . . . . 919
Fronczak, Francis E., Extraordinary Results in Treatment of Diphtheria 358

GARDNER, J. A., Genitourinary Cases . . . . . . . . 887
Genitourinary Cases . . . . 887
Gleet, Clinical Notes on. . . . . . 591
Goitre, Exophthalmic . . . . . 397
Greene, Walter D., Present Outbreak of Smallpox in Buffalo . . . . 492
Greenleaf, Clarence A., Therapeutic Value of the *X*-ray in Lupus Vulgaris . . . . . . . . 189
Grosvenor, J. W , Pemphigus 638
Guitéras, Ramon, Diagnosis and Surgical Treatment of Nephrolithiasis 709
Gunshot Wounds by Old and New Armaments . . . . . . 873
Gynecologic Diagnosis, Difficult Points in . . . . . . . . . . . 365

HAMANN, C. A., Spasmodic Torticollis and its Surgical Treatment . . . 321
Hanley, L. G., Labor Following Ventral Fixation 514
Ovarian Cyst, Hematoma and Fibroid . . . . 750
Removal of Two Large Pus Tubes . . . . 431
Report of St. Mary's Lying-in Hospital, 1901 . 579
Harrington, Devillo W., Practice of Medicine from a Business Standpoint . . . . . . . . . . . 18
Hartwig, Marcell, Charity by Proxy; Its Disastrous Effect upon the Physician. . . . . . . . . 22
Hats for Horses. . . . . . . . . . 62
Hauenstein, John, Comparative Methods of Vaccination, Old and New . . . . . . . 634
Heffron, John L., Science of Medicine and Healing Art . . 241
Hemorrhoids, Calomel in . . . 125
Heart Disease in Surgical Operations . . . . . . . . . . . 578
Hernia, Causes of Death in Strangulated . . . . . . . . 331
Heroin, Notes on Action of. . . . . 270
Treatment of Cough with 522
Hiccough, Treatment of Obstinate . 194
Himmelsbach, G. A., Normal Salt Solution by Hypodermoclycis . . 264
Hip-joint Disease . . . . . 87
Tubercular Disease of. . . 672
Hobbs, A. T., Pelvic Lesions; their Effects upon Mental Disturbances 473
Hopkins, Henry Reed, Fan System of Heating, Ventilating and Air Supply of Buffalo Schools. . 1
President State Medical Society . . . . 607
Report of the Committee on Hygiene. . . . . . . 565
Hospitals in Smaller Towns . . 184
Howe, Lucien, Frequency of Asthenopia, especially in America. . . . 117
Hygiene, Report of Committee on . 565
Hyperemesis, Gravidarum. . . . . 46

**Illustrations:**

Chart in President McKinley's Case . . . . . . . . 281
Clark, Edward, portrait . . . 528
Conover: Invalid Coach Service.
Fig. Ready to Receive Patient . . . 139
Evans: McK. and R. Nose Cup 677
Gihon, Albert Leary. Plate. . . 379

Page.

Gardner-Wilson: Genitourinary Cases.
Fig. 1, Stone, Natural size. . . . . . . . . 888
Fig. 2, Section of Stone . 889
Greene, Walter D., portrait . . 446
Hanley: Pus Tubes, Fig. . . 431
Hopkins, Henry Reed, portrait. . 607
LeBreton: Lateral Curvature of the Spine:
Fig. 1, Lateral Deviation of Spinous Processes, etc. . . . . . . . . . . 805
Fig. 2, Patient after Treatment in Pressure Machine. . . . . . 805
Fig. 3, Same Patient Showing Correction Treatment . 806
Fig. 4, Screw Pressure Machine . . . . . 809
Fig. 5, Mild Grade of Deformity . . 811
Fig. 6, Severer Type with Bony Changes. . 813
Fig. 7, Showing Structural Changes. . 815
Fig. 8, Showing Marked Rotation and Rigidity. 816
Fig. 9, Scoliosis in Friedreich's Ataxia . . 817
Moore, Edward Mott., portrait . 682
Moore, William A., portrait. . . 607
Pan-American Notes:
Fig. 1, Bonavita and His Group of Lions. . 148
Fig. 2, Wenona . 150
Fig. 1, Frank C. Bostock 235
Fig. 2, Bonavita's Masterpiece . . . 236
Fig. 3, Madame Morelli. . 236
Phelps: Tubercular Disease of the Spine and Hip.
Fig. 1, Specimen of Pott's Disease. . 86
Fig. 2. Specimen of Pott's Disease. . . 86
Fig. 3, Specimen of Pott's Disease. . 86
Fig. 4, Aluminum Corset 86
Hip-joint Disease.
Fig. 1, Improper Splint. . 90
Fig. 2, Cheap Dispensary Splint. . 92
Fig. 3, Inside Bar and Lateral Traction Splint 92
Fig. 4, Patent Splint, Adjustable High Shoe and Crutch . . . . . . . 93
Fig. 5, Double Dispensary Splint. . . . . . 94

PAGE.
Fig. 6, Side View of Fig. 4 . . 93
Fig. 7, Range of Motion after Treatment 91
Fig. 8, Anatomy of Hip-joint Muscles . 94
Fig. 9, Anatomy of Hip-joint Muscles 95
Fig. 10, Showing Patient Sitting with Splint . . 96
Potter: Century of Medical History:
Fig. 1, New German Hospital . . . . 504
Fig. 2, Fresh Air Mission Hospital. 506
Fig. 3, Pan-American Exposition Hospital . . . 507
President McKinley's Case:
Fig. 1, Shaking Hands with the President 209
Fig. 2, Where the President was shot . 211
Fig. 3, Roswell Park . . 212
Fig 4, Matthew D. Mann 213
Fig. 5, Herman Mynter. . 213
Fig. 6, John Parmenter. . 214
Fig. 7, Eugene Wasdin . 214
Fig. 8, P. M. Rixey . 215
Fig. 9, George B. Cortelyou . . . . 215
Fig. 10, Taking the President to the Exposition Hospital. . . 217
Fig. 11, Last Portrait of the President. 219
Fig 12, The President's last speech. 229
President William McKinley. Plate. . . . . 227
Professor Nothnagel, portrait . 854
Trowbridge, Josiah, plate, facing 525
Wende, Ernest, portrait . . . 448

INFANT INCUBATORS AT the Exposition. . 376
Insanity as a Defense in Criminal Cases. . . . . . . . . . . . 367

**Instruments, New.**

McKesson & Robbins Nose Cup . . . . . . . . . 676
Psychrophore, new—Dowd . . 885

**Items:**

Antikamnia Calendar, 1902 . . 551
Arlington Chemical Company's Group of Portraits . . . . 396
Arlington Chemical Company . 472

PAGE.
Army Medical Examinations . . 240
Campbell Private Sanitarium. . 551
Charles Roome Parmele Company, Removal of . . . . . 708
Clinical Records. . . . . . . . 80
Columbia Calendar, 1902 . . 551
Frank A. Ruf and Antikamnia . 471
Gude's Pepto-Mangan. . . . . 80
Indian Medicine . . . . . . . . 740
Infant Feeding . . . . 80
Medical History, County of Erie 552
M. J. Breitenbach Company. . 472
Mellier Drug Company . . . . 472
Parke, Davis & Company's great Industry at Detroit . . . . . 396
Parke, Davis & Company, Employees as Stockholders . 628
Phenacetin Patent Sustained (Farbenfabriken) . . . 708
Removals — Martin H. Smith Company, D. Appleton and Company . . . 860
Rio Chemical Company, Removal of . . . . . . 320
Rio Chemical Company . . . 472
Roberts Lymph Sanitarium 551
Sanguiform—Wyeth & Brother. 860
Scott & Bowne, Calendar, 1902 . 551
State Medical Examining and Licensing Board . . . . 160
Varoma Medical Company (Schieffelin). . . . . . . . 552

JEROME, District Attorney, of New York . . . . . 532
Jones, William B., Causes of Death in Strangulated Hernia 331

KIDNEYS, Anatomy of . . . . 24
Kidney, Technic of Fixation of Prolapsed . . . . 519
King, James E., Remarks on Sepsis and its Treatment . 553
Krauss, William C., Heredity, with Study of Statistics . . 892
Krönlein's Operation in Tumors of the Orbit . . . . . . . . . . . 34

LAGARDE, L. A., Gunshot Wounds by Old and New Armaments . . . . . . . . 873

**Leading Articles:**

American Public Health Association . . . . . . . . . . 136
Apropos of Osteopathy . 679
Assassination of President McKinley. . . . . . . . . . . 226

Page.
Automobile, the . . . . . . . . 375
Buffalo University Alumni Re-Union . . . . . . . . . . . 609
Buffalo University . . . . . . 840
Buffalo's New Health Commissioner. . . . . . . . 446
Coming Unification of the Medical Profession . 762
Coroner, Office of Abolished. . 767
Damages, Question of . . 768
Deputy Commissioner of Health 527
Dr. Wende's Retirement from Office. . . . . . . 447
Exhibits and Exhibitors at the Saratoga Meeting . . . . 913
Exposition, The, in its Medical Aspects . . . . . . . 130
Exposition, the, Farewell . . 297
Health Department of Buffalo . 373
Hospital, A Boarding. . . . . 448
Medical Association of Central New York. . . . . . . . . 233
Medical Department Pan-American Exposition. . . . . . . 445
Osteopathic Bill, the . . . . . 609
Passing of Sectarianism in Medicine . . . . . . . . . 298
Plea for Detention Hospital . . 49
President and Vice-president elect . . . . . . . . . . . 607
Quo Vadis ? . . . . . . . . . 914
Registration of Nurses . . . 845
Reorganised American Medical Association . . . . . . . . . 910
Report of the Physicians who Attended President McKinley 295
Semi-annual Meeting of the Medical Society State of New York . . . . . . 296
State License for Nurses . . . 678
State Medical Society in 1902 . 604
Streets, the . . . . . . . . . . 526
Substitution and Imitation . . . 843
Substitution and Imitation Again 918
Unification of the Medical Profession Again . . . . . . . 838
Union of Medical Societies in New York. . . . . . 442
Vital Statistics of the Census . 611
Wanted—An Isolation Hospital . . . . . . . . . . . 525
Voluntary National Examining Board . . . . . . . . . . 916

Le Breton, Prescott, Treatment of Rotary Lateral Curvature of the Spine. . . . . . . . . 804
Levien, Henry, Cough and its Treatment in Tuberculosis . . . . . . 110
Lewi, Maurice J., What Shall be done with the Professional Midwife ? . 563

Page.
Lewis, F. Park, Adenoids in Relation to Structural Changes. . . . . . 732

**Literary Notes:**

American Gynecological and Obstetrical Journal. . . . . 549
American Gynecology. . . . 938
American Journal of Medical Sciences. . . . . . . . . 395
Annual Report Buffalo General Hospital, 1901. . . . . . . 859
Atlas of Clinical Medicine (Blakiston) . . . . . . . . . 550
Battle's Anatomical Drawings, No. 5, 320, No. 6, 550, No. 7, 859, No. 8 . . . . . . . . . . 938
Brooklyn Medical Journal . . . 550
Buffalo State Hospital, 30th Annual Report. . . . . . . 471
Charity Eye, Ear and Throat Hospital . . . . . . . . . . 784
Cleveland Medical Journal . 627, 784
English Poetry Catalogue, Grosvenor Public Library . . . . 859
Enno Sander Prize . . . . . . . 320
Fox on Smallpox (Lippincott) . 708
Georgia Journal of Medicine and Surgery . . . . . . . . . . . 707
Gleason Sanitarium, Elmira . . 937
Homeopathic Journal of Pediatrics . . . . . . . . 471
Journal Association of Military Surgeons . . . . . . . . 471
Maltine Company's Prizes. . . 548
Nathan Lewis Hatfield Prize . . 626
Nicholas Senn Prize Medal . . 320
Nothnagel's Encyclopedia of Practical Medicine . . . . . 395
Optical News . . . . . . . . . 860
Physicians' Portraits and Biographical Sketches—Mariani. 859
Poisons and Antidotes (Maltine Co.) . . . . . . . . . . . . 937
Polk's Directory for 1902 . . . 938
Regional Minor Surgery—Van Schaick . . . . . . . . . . 784
Report of New York State Hospital for Crippled and Deformed Children . . . . . . . 859
Sanatoria for Consumptives—Dutton . . . . . . . 860
Southern California Practitioner 938
Syllabus of Bacteriology . . . 858
Vermont Medical Monthly . . . 626
Western Reserve University Bulletin . . . . . . . . . . . 471
William Wood & Company's Catalogue . . . . . . . . 938
Woman's Medical Journal . . . 626

PAGE.
Lupus Vulgaris, x-Fay in. . . . . . . 189
Lying-in Hospital Report, 1901 . . . 579

MALTINE Company's Prizes . 531
Mastoid Operation (Radical) with Technic . . . . . 785
Masturbation, Effects of . . . . . 891
McKinley, Details of President's Case . . . . 207
President, Official Report on the Case of—by P. M. Rixey, Matthew D. Mann, Herman Mynter, Roswell Park, Eugene Wasdin, Charles McBurney, Charles G. Stockton . . . . 271
President, Report on the Autopsy of, Harvey R. Gaylord . . . . . 284
President, Report on the Bacteriologic Examination of, Herman G. Matzinger. . . . 291
Surgery in the President's Case . . . . . . . . 205

McMichael, George H., Alcoholism, its Control and Cure in Sanatoriums . . . . . . . . . . 880
McMurtry, L.S., Treatment of Shock after Intra-abdominal Operations. 596
Meat, Substitution for 846
Medical College, Good Standing of. 63
History of the County of Erie . . . . . . . . 495
Inspection of Schools . . . 862
Mirror and the Reorganisation of the American Medical Association . . 137
Society County of Erie, Concerning Welfare of . 486
Medicine, Practice of From a Business Standpoint . . . 18
Science of and Healing Art 241
Mercury, its Action Upon the System 595
Midwife, What Shall be Done with the Professional . . . . . . 563
Milk Supply and Infectious Diseases 39
Modification . . . . . . . . 676

**Miscellany:**

Department of Health, Buffalo. 550
Laboratory Course in Bacteriology . . . . . . . . . . 628
Note on Albargin . . . . . . 627
Prize for Original Research in Epilepsy . . . . . . . . . 395

Moore, William A., Vice-President State Medical Society . . 608

PAGE.
Moore, Edward Mott, In Memoriam 900
Mycosis Fungoides, a Case of . . . 658
Mynter, Herman, Paraffin Injections 832

NASAL SEPTUM, Deviation of 655
Nephrolithiasis, Surgical Treatment of . . . 709
Nervous Cases, Treatment of Complicated . . . . . . . . . . 586
Nothnagel's System of Practical Medicine . . . . . . 848
Nurses as Sanitary Inspectors . . . 680
Nursing, Ethics of . . . . . . . . 251

**Obituary:**

Abbey, Emory C . . . . . . . 923
Babcock, Moses T . . . . 771
Bingham, Frank P . . . 141
Braman, Aaron Nelson . . . . 65
Brown, Charles Henry . . . 302
Castle, Frederick A. . . . . 850
Cochrane, Samuel R . . . . 922
Day, Dwight Washington . . 382
Dowd, Hepsabeth Marie . . . 850
Eastman, Joseph . . . . . . . 923
Eddy, John L . . . . . . . 770
Failing, Peter . . . . . . 617
Fenger, Christian . . . . . . 683
Fisher, Edward A . . . . . 683
Gihon, Albert Leary . . . 379
Gobrecht, William H . . . . . 65
Hay, Edward P . . . . . . . 141
Hayt, Charles W. . . . . . 141
Helmuth, William Tod . . . . 850
Homans, John, 2d . . . . . . 850
Jackson, Cassius O. . . . . . 617
Johnston, William Waring. . . 683
Jones, Leroy. . . . . . . 617
Keene, Joseph W . . . . . . 617
Kendall, James V. . . . 141
Lane, Levi Cooper . . . . . . 617
MacCormack, Sir William. . . 450.
Metcalfe, John T. . . . . . 617
Meyer, Jacob F . . . . . 922
Moore, Edward Mott . . . . . 681
Mundè, Paul Fortunatus . . . 615
Nichell, Henry, In Memoriam . 31
Parkhurst, Harvey . . . 617
Price, Eleazor . . . 617
Rhett, Robert Barnwell, Jr. . . 141
Rogers, Henry Raymond . . 302
Shepard, Jessie . . . . 142
Smith, William Merwin . . . . 534
Talcott, Selden H . . . . . . 923
Tilden, J. H. . . . . . . . . 923
Treat, William H . . . . . 617
Townsend, Morris W. . . . . 683
Upson, Anson Judd . . . . . 923
Whitcomb, Edwin E . . . . 141
Wight, Jarvis S . . . . . . . 382

PAGE.
OPHTHALMIA NEONATORum, Prevention of . . . 603
Ordinance, Anti-spitting . 532
Orthoform, Use of . . . . . . . 125
Osteopathy Bill Again . . . . . . 532
Otitis Media Neonatorum . . . . . 675
Ovarian Cyst, Hematoma and Fibroid 750

PAN-AMERICAN EXPOSItion, Medical Aspects of . . 53
Notes . . . 72, 148, 235
Report of Medical Department of. . . . 417
Pan-Am's Farewell. . . . . . . . . 376
Paraffin Injections . . . . . . . . 832
Park, Roswell, Report of Buffalo State Cancer Laboratory . 569
Report of the Medical Department of the Pan-American Exposition . . 417
Parmenter, John, Surgery in President McKinley's Case. . . . 205
Pelvic Lesions and Mental Disturbances. . . . . 473
Pelvic Suppuration, Suprapubic and Vaginal Methods of Treating . . 629
Pemphigus . . . . . . . . 638
Pensions, Commissioner of . . . . 532
Pericarditis, Tubercular. . . . . . 109
Peritonitis, Anodyne Treatment of Acute. . . . . . . . . . . . . 48

**Personal :**

Andrews, Robert M.; Murphy, J. B.; Urban, Adolph H.; Lothrop, Thomas; Park, Roswell . . . . . . . . . 681
Billings, Frank . . . . . 920
Blair, Alexander M.; McFarland, John S.; Haab, Prof. O . . . 922
Breuer, Max C.; Stoddart, James; Hoag, Myrtle A . . 850
Brown, Alice Barlow; Lydston, G. Frank; Elsner, Henry L.; Ricketts, Edwin; Dowd, J. Henry; Krauss, William C.; Potter, William Warren; Bosher, Lewis C . 533
Burrows, Lorenzo, Jr.; Hubbell, A. A.; Wright, Adam H. . . 63
Cleaves, Margaret A.; Gregory, Willis G.; Griffith, J.D.; Wood, Governor-General 65
Fox, Dr. and Mrs. Geo. Henry; Whitford, William . . . . 141
Gaylord, H. R.; McAdam, L. J.; Bodenbender, E. J.; Howard Charles F. . . . . . . . . 849
PAGE.
Hanley, L. G.; Hurty, J. N.; Rolph, R. T.; Ruffner, Ernest L.; Spalding, Thomas E.; Ullman, Julius; Whitmore, B. T. 64
Henry, Nelson H.; Gaylord, Harvey R.; Daggett, B. H. . 449
Hubbell, A. A.; Clausius, M. F. 302
Longyear, H. W.; Fowler, George Ryerson; Lewi, Maurice J.; Beach, Eugene; Suiter, A. Walter; Czerny, Vicerz; Leo-Wolf, C. G.; Cartledge, A.M.; Walker, Edwin; Price, Joseph; Macdonald, W. G.; Bacon, John E.; Haggard, William D., Jr.; Ruf, Frank A. . . . 301
McMurtry, L. S.; Henry, Nelson H . . . . . . . 769
Ricketts, Edwin; Stockton, Charles G.; O'Donnell, W. J.; Laidley, L.H.; Benedict, A.L.; Savage, Walter L. . . . 614
Riley, John W.; Doran, Robert E.; Wilson, Nelson W.; O'Donnell, W. J.; Mills, George F. . 450
Rixey, P. M.; Gihon, A. L.; Brush, George W. . . . . 300
Smith, Eugene; McMurtry, Lewis S.; Foster, Dr. and Mrs. William S.; Reed, Charles A. L.; Smiley, Alton L.; Coleman, N. R. . . . . . . . . . . . 140
Smith, Eugene; Putnam, James Wright; Jones, Allen A.; Roe, John O.; Ricketts, Edwin; Mynter, Herman; Dunham, S. A.; Cutting, Reger; Borzelleri, Charles R.; Parker, W. E . . . . . . . 921
Southall, Edward A.; Elsner, Henry L.; Clayton, Mary; Smith, Edward T. . . 615
Stockton, Charles G.; Meyer, Edward J.; Persons, A. E.; Park, Roswell; Mills, G. F.; Russell, Nelson G.; Leo-Wolf, C. G.; Pryor, John H.; Harris, John T. . . . . . . . . . 770
VanPeyma, P. W. . . . . . . 534
Wilson, Nelson W . . . . . . 377
Waddell, Mr. "Doc."; Thomas, T. Gaillard; Kelly, Catherine E.; Danser, Earl G.; Bulkley, L. Duncan; Burrows, Lorenzo Jr.; Chester, Charles O . . . 378

Phelps, A. M., Tubercular Disease of the Spine and Hip . . . . . 81
Phelps, William C., Concerning the Welfare of the Medical Society County of Erie . . . . . . . . 486

PAGE.

Pleurisy, Purulent . . . . . . . . 125
Pneumonia in Children, Treatment of 38
Potter, J. H., Consideration of Functional Signs of Diseases of the Stomach . . . . . . 341
William Warren, Century of Medical History in the County of Erie . . . . . 495
Potts' Disease . . . . . . . . . . 81

**Progress in Medical Science:**

Genitourinary and Syphilitic Diseases, by Byron H. Daggett . . . . 42, 519, 591, 758
Neurology, by Wm. C. Krauss. 367
Obstetrics, Gynecology and Abdominal Surgery, by William Warren Potter. . . . 363
Ophthalmology, by Alvin A. Hubbell. . . . . . . . . 34
Orthopedic Surgery, by Prescott Le Breton. . . . . . . 672
Pediatrics, by Maud J. Frye, 37, 674
Therapeutic Notes, by Nelson W. Wilson . . . . . . . . . 124

Prostatic Disease, Treatment of by Hot Solution . . . . . 884
Hypertrophy . . . . . . 12
Pruritus Ani. . . . . . . . . . . . 819
Pryor, W.R., Suprapubic and Vaginal Methods of Treating Pelvic Suppuration. . . . . . . . . . . 629
Pus Tubes, Removal of Two Large. 431
Pyelitis, Treatment of, in Women . 363

**Reviews:**

Abbott: Hygiene of Transmissible Diseases . . . . 459
Abel: Gynecological Pathology. 690
Anders: Practice of Medicine . 464
Bergey: Principles of Hygiene. 542
Bishop: Uterine Fibromyomata 462
Blakiston: Physician's Visiting List for 1902 . . . . . . 390
Blakiston: Three Thousand Five Hundred Questions on Medical Subjects . . . . . . . . 391
Boas: Diseases of the Intestines 456
Brower: Manual of Insanity . . 928
Brubaker: Compend of Human Physiology . . . . . . . . 79
Bruhl: Atlas of Otology . 930
Bryant: Operative Surgery, Vol. II. . . . . . . . . . . . . 459
Bulkley *et al.*: Symposium on Syphilis. . . . . . . . . . 783
Budgett: Essentials of Physiology . . . . . . . . . . . . 705
Buck: Reference Handbook of the Medical Sciences, Vol. III. 460
Vol. IV. . . . . . 931
Bürck: Atlas and Epitome of Pathologic Histology, Vol. II. 625
Burdick: Handbook of Practical Medicine . . . 621
Burnett-Ingals: Diseases of Ear, Nose and Throat. 777
Butler: Diagnostics of Internal Medicine . . . . . 455
Cabot: Clinical Examination of the Blood. . 386
Cattell: International Clinics, Eleventh Series, Vol. II., 387; Vol. III., 623; Vol. IV. . . . 702
Cheyne-Burghard: Manual of Surgical Treatment, Vol. V. . 310
Church-Peterson: Nervous and Mental Diseases . . . . 543
Coakley; Diseases of Nose, Throat, etc. . . . . . . 388
Cohen: System of Physiologic Therapeutics, Vols. I. and II., 157; Vols. III. and IV., 457; Vol. VI. 780
Corlett: Acute Infectious Exanthemata. . . . . . 536
Craig: Malarial Fevers . . . 464
Crothers: Morphinism and Narcomania . . . 927
Crothers: Drug Habits and their Treatment . . . . . . 933
Dana: Nervous Diseases . 704
Davenport: Manual of Gynecology. . . . . 935
Delafield: Pathologic Anatomy and Histology . . 463
DeLaurence: Hypnotism . . . 238
Dorland: Medical Dictionary . 461
Dorland: Modern Obstetrics 699
Dowd: Specific Urethritis, Treatise on . . . . . . . 314
Eichhorst: Practice of Medicine 315
Ellis: Psychology of Sex, Vol. II. 782
Engelhard: Standard Medical Directory . . . . 624
Estes: Treatment of Fractures 236
Ewing: Clinical Pathology of the Blood. . . . . . . 311
Fischer: Infant Feeding 546
Fogge: Pocket Gray. . . . . 934
Galbraith: Four Epochs of Woman's Life. . . 542
Gaylord - Aschoff: Pathological Histology . . . 687
Goelet: Surgical Gynecology . 703
Gottheil: Syphilis, its Diagnosis and Treatment. . . 388
Gould: Year-Book of Medicine and Surgery, 1902 . . . . . 782

PAGE.
Gray: Anatomy, 8th Edition . 781
Haab: Atlas of Ophthalmoscopy 153
Hackett: Venereal and Sexual Diseases 620
Hare: Progressive Medicine, Vol. II., 1901, 158; Vol. III., 456; Vol. IV., 624; Vol I, 1902 . . . . . . 856
Harrington: Practical Hygiene. 312
Hatfield: Contagious Diseases of Childhood . . 158
Hayden: Venereal Diseases. . 781
Head: Practical Medicine Series,
Vol. I., General Medicine 696
Vol. II., General Surgery 701
Vol. III., Eye, Ear, Nose and Throat . . . . 930
Hektoen-Riesman: Text - Book of Pathology . . . . . . 539
Hemmeter: Diseases of the Intestines . 776
Hirst: Text-Book of Obstetrics 625
Hollander: Mental Functions of the Brain. . . 691
Holmes: Outlines of Anatomy. 695
Jackson: Handbook of Skin Diseases . . . . . 385
Jackson: Refraction and Diseases of the Eye 465
Jakob: Atlas of the Nervous System . . . . . 76
Jennings: Manual of Ophthalmoscopy . . . . 934
Jewett: Practice of Obstetrics. 699
Jullien: Libertinism and Marriage . . . . 775
Keersmaecker - Verhoogen: Chronic Urethritis, etc. . . 77
Kehr: Gallstone Disease 315
Keysor: Medico-Legal Manual 238
Lea: Medical News Visiting List for 1902. . . . 392
Lehmann-Neumann: Atlas and Epitome of Bacteriology, Vols. I. and II. . 619
Leuf: Practical First Principles 927
Lockwood: Practice of Medicine . . . . 700
Mallory: Pathological Technic. 466
Mariani: Celebrities from Album of . . . . 316
May: Diseases of the Eye . 463
Mays: Consumption, Pneumonia and Allied Diseases 312
McFarland: Pathogenic Bacteria . . . 544
Mortimer: Peru History of Coca 778
Nancrede: Principles of Surgery, Lectures . . . 313
Neff: Manual of Prescription Writing . . . . . . . . . 934

PAGE.
Nichols: Clinical Laboratory Methods . . . 855
Nothnagel: Encyclopedia of Medicine, Vols. I. and II. . . 853
Osler: Principles and Practice of Medicine . . . . 461
Packard: History of Medicine in the United States . . 703
Park: Treatise on Surgery. . . 694
Penrose: Diseases of Women . 543
Pershing: Nervous and Mental Diseases. . . . . 932
Potter: Materia Medica, Pharmacy and Therapeutics . . 387
Powell: Essentials of Diseases of Children . . . . . . 79
Price: Handbook on Sanitation 389
Raymond: Human Physiology . 700
Reports:
Commissioner of Education 1899 and 1900 . 704
Illinois State Board of Health 460
Johns Hopkins Hospital, Vol. VIII., 1900, Typhoid Fever, 239; Vol. X., 1901, Malarial Parasites, etc. . 317
Medical Directory, New York, New Jersey, and Connecticut . . . 468
Michigan State Board of Health, 1900 and 1901 . 468
Mt. Sinai Hospital, Vol. II., 1899 and 1900 . . . . 693
New York State Board of Health, 1899. . . . . . 545
Roberts: Deformities of the Face . . . 705
Rotch: Pediatrics . . . . . 467
Sajous: Cyclopedia of Practical Medicine, Vol. VI. 79
Schaeffer: Atlas of Labor and Operative Obstetrics 154
Schaeffer: Atlas of Obstetric Diagnosis and Treatment 154
Schamberg: Diseases of the Skin . . . 545
Scudder: Treatment of Fractures . . 159
Senn: Principles of Surgery. . 313
Senn: Practical Surgery. 458
Shield: Lectures on Nasal Obstruction . . . . . . . . . 390
Shoemaker: Diseases of the Skin 465
Shoemaker: Materia Medica and Therapeutics . . . . 932
Simon: Guide for Beginners in Chemistry. 622
Simon: Manual of Chemistry . 467

PAGE.

Simon: Manual of Clinical Diagnosis . 929
Simon: Physiological Chemistry 697
Sollman: Text-Book of Pharmacology . . . . 779
Starr: Digestive Diseases of Childhood. . . 390
Sternberg: Text-Book of Bacteriology . . . 546
Strümpell: Text-Book of Medicine . . 698
Tanner: Memorandum of Poisons . . 388
Taylor: Physician's Pocket Account Book . . . 391
Thayer: Compend of Pathology 935
Thorington: Retinoscopy 389
Thornton: Dose-Book and Prescription Writing. 704
Thornton: Pocket Formulary .. 933
Tillmans: Text-Book of Surgery . . . . . . . . . 548

Transactions:

American Association of Obstetricians and Gynecologists, Vol. XIII., 1900 159
American Dermatological Association, 1900. 386
American Laryngological Association, 1900. 469
American Laryngological Association, 1901 . 933
American Surgical Association, 1901. . 856
Medical Association of Central New York 469
Medical Association of Alabama . 706
Medical Association of Texas. . . 706
Medical Society State of New York, 1901 468
Mississippi Valley Medical Association, Vol. II., 1900 316
Southern Surgical and Gynecological Association, Vol. XIII., 1900 316
Wisconsin Medical Society 935

Treat: International Medical Annual 779
Treves: Surgical Applied Anatomy. . . 466
Tyson: Physical Diagnosis . 705
Vecki: Sexual Impotence. . . 462
Wainwright: New Remedies and Therapeutic Measures. 314
Warwick-Tunstall: First Aid to the Injured . . . . . . 544
Waugh: Diseases of the Respiratory Organs . . . . . . . 391

PAGE.

Whitman: Orthopedic Surgery 237
White: Materia Medica, Pharmacy, etc.. . . . . 625
Williams: Manual of Bacteriology . . . . 702
Winters: Feeding of Infants. . 79
Wolf: Physiologic Chemistry . 545
Wood: Medical Record Visiting List for 1902 . . . . 391
Zuckerkandl: Atlas of Operative Surgery . . . . . . . . 931

RENAL DISEASES, Treatment of. . . 42
Renner, W. Scott, Radical Mastoid Operation, with Technic.. . . . 785
Rochester, DeLancey, Medical Inspection of Schools . . . . . . 862
Ryerson's Solution . . . . . . . . 204

SALT SOLUTION, Formula for Normal . 127
Normal by Hypodermoclycis . . 264
Sargent, George W., Carbolic Acid, its Use and Abuse . . . 337
Scabies in Children. . . 126
Scarlet Fever, Deafness from 247
Schools, Heating, Ventilating and Air Supply of . . . . . 1
Sepsis and its Treatment 553
Shock of Intra-abdominal Operations . 596
Sloane, W. Harpur, The best Alkaline Wash. . . . . . . . 357
Smallpox in Buffalo . . 377
Present Outbreak of. . . 492
Relation of to Swine. . . 613
Smith, Eugene A., Relation Between Dentist and Surgeon 826
Snow, Sargent F., Deafness from Scarlet Fever . . . . . . . . 247

**Society Meetings:**

American Academy of Medicine 925
American Association of Obstetricians and Gynecologists . . . . . 65, 145, 233, 924
American Association of Pathologists and Bacteriologists 684
American Association of Urologists . . . 684
American Congress of Tuberculosis. . . . . . . 684, 771
American Electro-therapeutic Association . . . . 67, 147, 306, 771
American Medical Association . 771

PAGE.

American Orthopedic Association. . . . . . . . . . . 853
American Public Health Association . . . . . . . . 69, 143, 307
American Surgical Association . 851
American Urological Association . . . . . . . . . 925
Association of American Medical Editors . . . . . . . 143
Association of Military Surgeons . . . . 851, 924
Buffalo Academy of Medicine, . . . 68, 234, 303, 308, 383, 452, 535, 618, 684, 772, 852, 925
Egyptian Medical Congress 384
Erie County Medical Association 852
Erie Railway Surgeons Association. . . . . . . . . 308
Esculapian Club . . . . 452
Fourteenth International Medical Congress. . . . . 306
German Naturalists and Medical Congress . . . . . 235
Harvard Medical Society of New York . . . . . 618
Homeopathic Medical Society of the State of New York, 67, 309
International Association of the Medical Press . . 684
Lake Keuka Medical and Surgical Association. . . . . 70
Lake Erie Medical Society, 309, 53
Medical Association of Central New York . . . . . . 69, 143
Medical Society of Missouri Valley . . . 68, 771
Medical Society County of Chautauqua . . . . . 68, 384, 452
Medical Society County of Cattaraugus . . . . . . . . 142
Medical Society State of New York . . . . . . . 235, 451
Medical Society of the County of Erie . . . . . . . . . . 851
Medical Union. . . . . . . . . 535
Mississippi Valley Medical Association. . . . . . . 142, 308, 771
National Association of Homeopathic Medical Examining Boards . . . 67
National Confederation of State Medical Examining and Licensing Boards. . . . . . 853
New York State Medical Association . . . . . . . 309
Niagara Falls Academy of Medicine. . . . . . . 384, 534, 772
Missouri State Medical Association . . . . . . . . . . . 686, 772
Physicians' League . . 618, 851
Rochester Academy of Medicine 853

PAGE.

Society of Physicians of Canandaigua . . . . . . . . . . . 685
Southern Surgical and Gynecological Association . . . 143, 383
Thomas J. King Medical Club . 142
United States Pension Examiners' Association . . . . . 925
U. S. Pension Examiners of New York . . . . . . . . . 851
Western Ophthalmologic and Oto-laryngologic Association 685
Western Surgical and Gynecological Association . . . . . 451

**Society Proceedings:**

Alumni Association, University of Buffalo . . . . . . 902
British Congress of Tuberculosis 34
Buffalo Academy of Medicine, . . . 123, 203, 359, 667, 670, 899
Section on Ophthalmology, Otology, Rhinology and Laryngology 123, 360
Section on Pathology 302, 432, 588, 669, 751
Section on Obstetrics . . 667
Section on Surgery 671
Buffalo Ophthalmological Club. 757
Medical Association of Central New York . . . . . 196
Medical Society of the County of Erie . . . . . . 29, 515
New York Medical Jurisprudence Society 434
Roswell Park Medical Club, 361, 433

Spine, Rotary Lateral Curvature of . 804
Spratling, Wm. P., Plea for Broader Treatment of Epilepsy . . . . . 161
Sternberg, George M., Dinner to . . 920
Promotion for Surgeon-General . . . . . 847
Stomach, Functional Signs of Diseases of. . . . . . . . . . . . 341
Stone in the Bladder, Large . . . . 887
Substitution in Physicians' Prescriptions . . . . . 299
Suprarenal Extract, on the Action of 36
Syphilis, Curability of . . . . . . 758
Proper use of Mercury in . 438

TEST, The Two Glass . . . . . 181
Torticollis, Spasmodic, Treatment of . . . . . . . . 321
Tubercular Disease of the Spine and Hip. . . . . . . . . . 81
Tuberculosis, Cough and its Treatment in . . . . . 110
Renal and Bladder . . 482

PAGE.
UTERUS, Fibroid Tumors of... 365
Uterine Appendages, Conservative Treatment of..... 366

VACCINE VIRUS, Commercialism and Responsibility in Connection with ..... 434
Vaccination, Comparative Methods of .............. 634
Valyl.............. 759
Venereal Diseases, Relation of Evolution to Infection in ...... 589
Ventral Fixation, Labor Following . 514
Virchow, Anniversary, The .... 300
Volcanic Eruptions in Windward Islands............... 847

PAGE
WASH, The Best Alkaline 357
Wende, Ernest, Dinner to ...... 529
Wilson, Nelson W., Details of President McKinley's Case 207
Duties of, as Sanitary Officer of the Exposition . 137
Genitourinary Cases . . . 887
Indian Medicine . . 740
Wood, Casey A., Exophthalmic Goitre; Its Etiology, Symptoms and Treatment.... . 397
Wynkoop, Edward Judson, Choice of an Anesthetic for Children . . 168

YOUNG, A. A., The Vermiform Appendix and Appendicitis. 257

